# Comparative evaluation on the bond strength of AH PLUS sealer to root dentin after using different gutta-percha solvents during retreatment

**DOI:** 10.3389/fdmed.2026.1953363

**Published:** 2026-09-29

**Authors:** Shravan Kini, Kishan Paul Raja, S. Vidhyadhara Shetty, Mohammed Aleemuddin, Jayaprakash K

**Affiliations:** 1 Department of Conservative Dentistry and Endodontics, Yenepoya Dental College, Yenepoya (Deemed to be University), Mangalore, Karnataka, India; 2 Department of Dental Materials, Biomaterials and Research Center, Yenepoya Dental College, Yenepoya (Deemed to be University), Mangalore, Karnataka, India

**Keywords:** AH plus sealer, essential oils, gutta percha, push out bond strength, retreatment

## Abstract

Complete removal of gutta-percha during endodontic retreatment is essential for effective canal disinfection and resealing of the root canal system. Gutta-percha solvents may alter dentin characteristics and influence adhesion of sealers. This *in vitro* study evaluated the effect of castor oil, peppermint oil, wintergreen oil, and xylene on the bond strength of AH Plus sealer to root dentin. Forty extracted human single-rooted teeth were prepared, obturated, and randomly assigned to four groups (*n* = 10) based on the solvent used during retreatment. Following removal of the root canal filling material and re-obturation with AH Plus sealer, one 1-mm-thick root section was selected from each tooth for push-out bond strength testing using a universal testing machine. Data were analyzed using one-way ANOVA and Bonferroni *post hoc* tests. Significant differences were observed among the groups (*p* < 0.001). Castor oil demonstrated the highest bond strength, followed by wintergreen oil, xylene, and peppermint oil. Wintergreen oil showed bond strength values comparable to xylene, whereas peppermint oil exhibited the lowest values. Within the limitations of this study, the choice of gutta-percha solvent significantly influenced the bond strength of AH Plus sealer to root dentin. Castor oil showed superior performance, while wintergreen oil may be considered a potential alternative to xylene.

## Introduction

The root canal treatment proves effective primarily due to thorough cleaning and shaping of the root canal system, in addition to perfect three-dimensional obturation of it. The failure to do so properly can cause microorganism persistence in the root canal system, leading to adverse consequences after the treatment procedure (1). Endodontic retreatment refers to the process whereby an already treated tooth showing symptoms of inflammation is retreated again to rid it of bacteria that may persist in the tooth structure. In such a case, it becomes necessary to remove previously filled materials in the tooth like the gutta-percha, thus allowing access to the bacteria. Several techniques have been utilized in the removal process, including stainless steel hand files, nickel-titanium rotary instrument, heating method, and ultrasonic waves (2). Additionally, the employment of a solvent during the procedure is suggested to facilitate the removal process since it makes the gutta-percha soft (3).

The reason why GP removal during endodontic retreatment is a difficult procedure is based on the unique properties of thermoplasticity, density, and elasticity of GP that allow it to fit tightly into the root canal system, including its lateral parts and dentinal tubules. The combination of GP along with cements such as AH Plus leads to strong mechanical adhesion between the cements and dentin, as well as between cement and GP, resulting in high shear and push-out bond strength values (approximately 2-5 MPa). Additionally, shrinkage of the cements along with the elasticity of GP creates resistance to removal through friction locking (4).

The use of xylene, which is an aromatic hydrocarbon, in the process of removing gutta-percha is due to its ability to soften the gutta-percha. This is because it softens gutta-percha by making it less rigid and adhesive. In other words, xylene works by making gutta-percha easy for dental instruments to remove it (5). However, it has been noted to be toxic, and irritating to the periapical region. Besides, when ingested or breathed in large quantities, xylene becomes dangerous, posing threats to one's heart, lungs, and liver (6, 7). The use of safer alternatives such as orange oil has been observed to have several advantages, especially with regard to biocompatibility and dissolution of root canal sealer materials (8).

Contact between gutta-percha solvent and tooth structure can cause chemical changes in coronal and radicular dentin tissues in the course of retreatment procedure (9, 10). Residual Chloroform has already shown its ability to reduce the bonding effectiveness of sealers (Resilon/Epiphany SE, AH Plus, Sealapex, and others) (11). Taking into account mentioned above facts, natural oils (castor oil, peppermint oil, wintergreen oil, etc.) are gaining more attention nowadays because of their high biocompatibility compared to artificial solvent. However, modern literature does not provide any consensus on influence of solvents on dentine properties and adhesion of sealer material. Systematic reviews suggest that while natural solvents are biocompatible, their overall effect on dentin properties and subsequent sealer bond strength can be highly variable (12). Currently, available data concerning the influence of solvents on root dentin bond strength of epoxy-based sealers (AH Plus, etc.) are still very limited. Therefore, current study is aimed to determine and compare bond strength of AH Plus sealer to root dentin in case of using different types of gutta-percha solvents (xylene, castor oil, peppermint oil, wintergreen oil).

## Materials & method

This *in vitro* study was carried out on 40 intact human permanent single-rooted premolars from both the maxilla and mandible. The teeth were extracted for therapeutic reasons and collected from the Department of Oral and Maxillofacial Surgery, Yenepoya Dental College, Mangalore, Karnataka, India. The teeth were obtained after extraction and were used for the present *in vitro* study following the applicable institutional ethical requirements. Each tooth was meticulously inspected under a dental operating microscope to detect any cracks, root fractures, or structural abnormalities, and those showing such defects were excluded from the study.

The selected specimens were first cleansed to eliminate blood and debris, and then immersed in 5.25% sodium hypochlorite for a duration of 24 h. Residual periodontal tissue and calculus were subsequently removed using a scaler. Following cleaning, the teeth were stored in normal saline at room temperature until they were utilized for further procedures.

Access cavities were prepared using a sterile No. 2 round bur attached to a high-speed contra-angle handpiece under ample water irrigation. The pulpal tissue was removed with a barbed broach, after which canal negotiation and patency were confirmed using a No. 15 K-file. The working length was determined radiographically and set at 0.5 mm short of the apical foramen.

Mechanical instrumentation was carried out using ProTaper Gold rotary files (Dentsply Sirona, Ballaigues, Switzerland) up to size F3, employing a crown-down approach. During the cleaning and shaping process, abundant irrigation was performed with 5.25% sodium hypochlorite and 17% ethylenediaminetetraacetic acid (Prevest DenPro Ltd., Jammu, India), used alternately.

After instrumentation, the canals were given a final rinse with normal saline and then dried using sterile paper points. Obturation was performed using gutta-percha along with AH Plus sealer (Dentsply Sirona, Konstanz, Germany). The filled specimens were subsequently stored at 37 °C in an environment of 100% humidity for two weeks.

Subsequently, the obturated teeth were randomly allocated into four groups (*n* = 10 per group) using a computer-generated randomization sequence based on the type of solvent used.

Group 1 – Castor oil group (*n* = 10)
Group 2 – Peppermint Oil (*n* = 10)
Group 3 – Wintergreen oil (*n* = 10)
Group 4 – Xylene (*n* = 10)

Xylene was included as the conventional comparator/reference solvent, against which the three natural oil-based solvents (castor oil, peppermint oil, and wintergreen oil) were compared.

Castor oil (Khadi India, Swadeshi Herbal; 100% pure, cold-pressed), peppermint oil (Naturalis, Mentha piperita; 100% pure essential oil, 30 mL), and wintergreen oil (Swastik, Gaultheria; 100% pure essential oil) were used as the natural oil-based solvents. Xylene was used as the conventional solvent. All solvents were obtained commercially and the same source was used throughout the study.

For each experimental group, two drops (approximately 0.1 mL) of the respective solvent were applied to the canal orifice using a 27-gauge syringe and allowed to act for 2 min. No further reapplication of the solvent occurred during the mechanical retreatment procedure. Coronal gutta-percha was then removed using Gates-Glidden drills (#1, #2, and #3; Mani Inc., Tochigi, Japan).

Subsequently, ProTaper retreatment rotary files were used to mechanically engage and remove the softened gutta-percha from the canal walls using a crown-down technique toward the apex. Complete gutta-percha removal was verified visually and tactilely under the dental operating microscope and confirmed radiographically prior to re-obturation. The same application time, solvent quantity, and mechanical removal protocol were used for all four groups.

Finally, all specimens were retrofilled using F3 gutta-percha cones and AH Plus sealer (Dentsply Sirona, Konstanz, Germany) through the modified lateral condensation method. The access cavity was then filled and the samples placed in an environment of 37 °C and 100% humidity for two weeks.

After the period of storage, each tooth was placed in chemically cured acrylic blocks in a vertical manner. Each root was sectioned horizontally into 1-mm-thick sections corresponding to the coronal, middle, and apical thirds of the root using a low-speed diamond precision saw.

One 1-mm-thick section was selected from each tooth for push-out bond strength testing, resulting in 10 tested specimens per experimental group (Figure 1). To prevent inconsistent selection from confounding the results, a standardized and fixed distribution of root levels was assigned to each group. Specifically, within every group of 10 specimens, 3 sections were chosen from the coronal third, 3 from the middle third, and 4 from the apical third. This identical distribution was maintained across all four experimental groups to ensure that root depth was consistently controlled.

**Figure 1:**
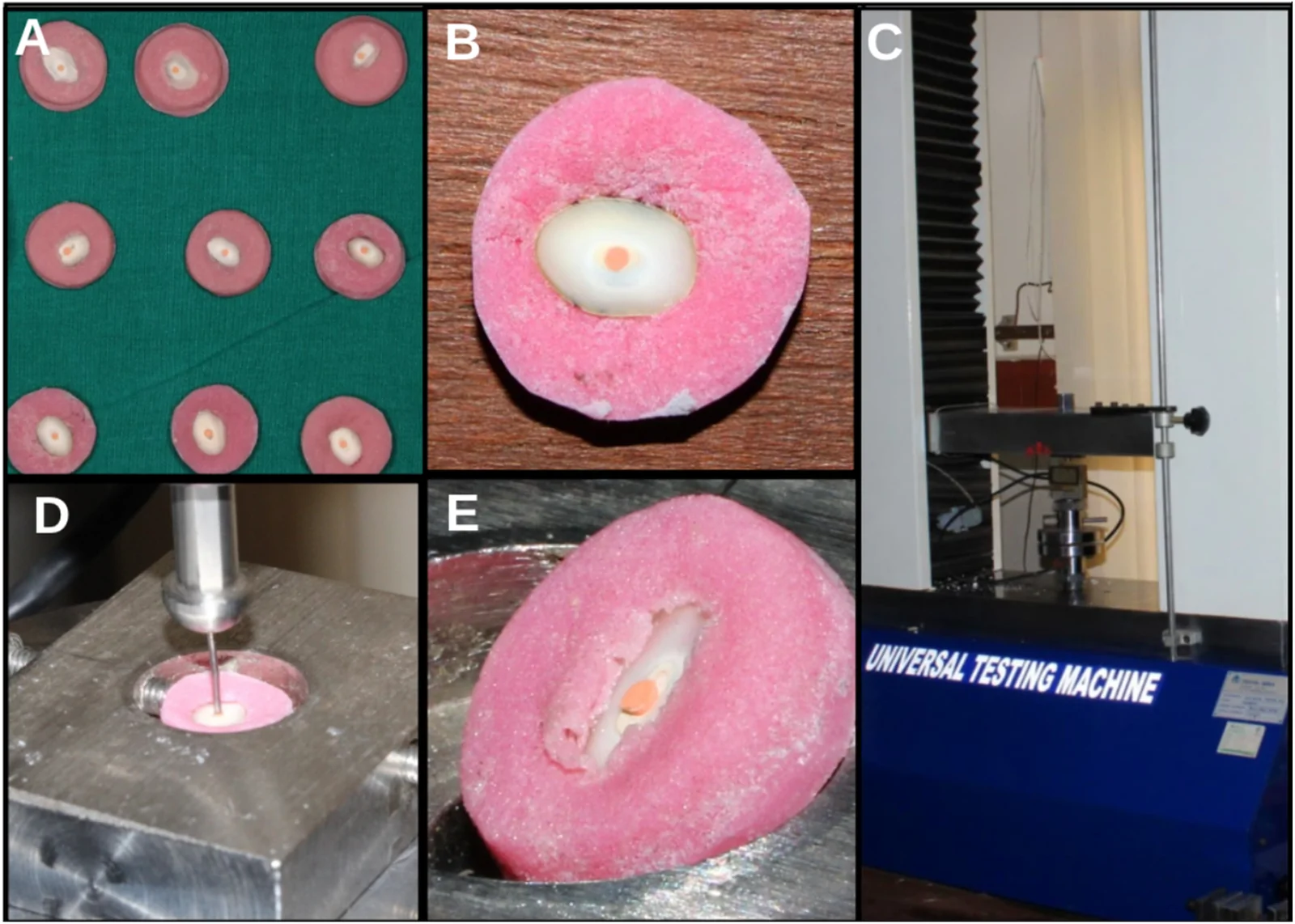
Push-out bond strength testing procedure. **(A,B)** Mounted cross-sections of the obturated tooth. **(C)** Universal Testing Machine. **(D)** Placement of the mounted tooth in the universal testing machine. **(E)** Extrusion of the gutta-percha following push-out bond strength determination.

Because the primary objective of the study was to compare the effect of the four gutta-percha solvents on the overall push-out bond strength of AH Plus sealer to root dentin, the root levels were solely controlled to eliminate bias and were not treated as independent experimental variables for statistical comparison.

The specimens were placed on a jig with a central cavity for conducting tests in a Universal Testing Machine (Tecsol-India). A customized cylindrical stainless-steel plunger (0.8 mm diameter) was used to apply a compressive load to the obturation material at a crosshead speed of 1 mm per minute, avoiding contact with the surrounding dentin.

Prior to push-out testing, the exact radius of the obturated canal for each 1-mm section was measured using a digital caliper to account for anatomical taper. The average radius was then applied to the formula Adhesion Area = 2πrh.

The bond strength was then calculated using the below formula:

$$\mathrm{Bond}\;\mathrm{Strength}\;(\mathrm{MPa})\;=\;\mathrm{Maximum}\;\mathrm{Load}\;(N)/\mathrm{Adhesion}\;\mathrm{Area}\;(mm^{2})$$

AdhesionArea=2πrh(r=radiusofcanal,h=1mm)

## Sample size calculation

The sample size was calculated using G*Power software based on a one-way ANOVA with four groups. Although the initial *a priori* calculation (based on a pooled standard deviation of 0.37, an expected large effect size of f = 0.838, a 5% level of significance, and 99% statistical power) indicated a smaller minimum requirement, the final sample size was conservatively set to 10 specimens per group (total *N* = 40). This larger sample size was deliberately established to maximize statistical robustness against inherent anatomical variations in natural human teeth, ensure adequate statistical power is maintained across all group comparisons, and preemptively account for any potential specimen loss during the precise horizontal root sectioning procedures.

## Statistical analysis

The obtained results were analyzed using Microsoft Excel and IBM SPSS Statistics 27 software. In order to determine the normality of distribution of the data, the Shapiro–Wilk test was used. It was shown that the distribution of the data had a normal distribution. The intergroup comparisons between four groups were conducted using one-way ANOVA with Bonferroni correction for multiple testing. The *p*-value of <0.05 was statistically significant. The statistical analysis was performed to compare push-out bond strength among the four solvent groups. Root level (coronal, middle, and apical) was not included as an independent factor because comparison among root levels was not an objective of the present study.

To ensure objectivity and minimize bias, blinding was implemented during the testing and data analysis phases. The examiner who performed the push-out bond strength tests using the Universal Testing Machine and the statistician responsible for data evaluation were kept blind to the specific solvent group allocations throughout the experimental procedures.

## Results

The push-out bond strength values (mean ± standard deviation) recorded for the four experimental groups were as follows: Castor oil group demonstrated the highest mean bond strength (5.88 ± 0.24 MPa), followed by wintergreen oil (5.23 ± 0.19 MPa), xylene (4.89 ± 0.56 MPa), and peppermint oil (4.48 ± 0.16 MPa).

Different mean values were recorded for the four groups; the highest mean value was observed in the Castor group followed by Wintergreen, Xylene, and Peppermint respectively. The greatest variation was in the Xylene group while the lowest was in the Peppermint group. (Table 1)

**Table 1:** Descriptive statistics for push-out bond strength across solvent groups.

Solvent groups	Mean	Std. Deviation	Std. Error	95% Confidence Interval for Mean		Minimum	Maximum	Test statistic	p value
				Lower Bound	Upper Bound				
Castor	5.8830	0.23889	0.07554	5.7121	6.0539	5.50	6.23	32.807	<0.001
Peppermint	4.4760	0.15693	0.04963	4.3637	4.5883	4.23	4.73		
Wintergreen	5.2300	0.18625	0.05890	5.0968	5.3632	4.93	5.47		
Xylene	4.8920	0.56109	0.17743	4.4906	5.2934	4.33	5.92		

One-way ANOVA showed that there was a significant difference between the four groups (F(3, 36) = 32.807, *p* < 0.001, partial \eta^2 = 0.732). However, because root-third distribution was not treated as a statistical covariate, these pooled overall means should be interpreted cautiously regarding inherent tubule-density variations.

The *post-hoc* Bonferroni analysis demonstrated statistically significant differences in several pairwise comparisons among the solvent groups. Specifically, Castor exhibited statistically significantly greater mean values than those in Peppermint (mean difference = 1.407, 95% CI: 0.997 to 1.817, *p* < 0.001), Wintergreen (mean difference = 0, 95% CI: 0.243 to 1.063, *p* < 0.001), and Xylene (mean difference = 0.991, 95% CI: 0.581 to 1.401, *p* < 0.001). Peppermint had significantly smaller mean values than Wintergreen (mean difference = −0.754, 95% CI: −1.164 to −0.344, *p* < 0.001) and Xylene (mean difference = −0.416, 95% CI: −0.826 to −0.006, *p* = 0.045). Wintergreen and Xylene were not found to be statistically different (mean difference = 0.338, 95% CI: −0.072 to 0.748, *p* = 0.163). (Table 2)

**Table 2:** Post-hoc Bonferroni multiple comparisons between solvent groups.

(I) Group	(J) Group	Mean Difference (I-J)	p value	95% Confidence Interval	
				Lower Bound	Upper Bound
Castor	Peppermint	1.40700^*^	<0.001	0.9970	1.8170
	Wintergreen	0	<0.001	0.2430	1.0630
	Xylene	.99100*	<0.001	0.5810	1.4010
Peppermint	Wintergreen	−0.75400*	<0.001	−1.1640	−0.3440
	Xylene	−0.41600*	0.045	−0.8260	−0.0060
Wintergreen	Xylene	0.33800	0.163	−0.0720	0.7480

*indicates a statistically significant difference (*p* < 0.05).

## Discussion

In the case of non-surgical retreatment of teeth previously treated endodontically, removal of all gutta-percha and sealer materials from within the canal system is critical. It makes way for easier access to the apical part of the canal system, which provides an opportunity for proper disinfection and sets up favorable conditions for effective obturation (13). Removal of GP material in the process of endodontic retreatment can be accomplished through a wide range of methods, which include mechanical instrumentation, thermal procedures, and the use of chemical solvents that melt or dissolve GP material. The most commonly used chemical solvents include chloroform, xylene, and halothane, which are considered highly effective and efficient. However, they are associated with several disadvantages related to cytotoxicity and potential harm to the periradicular tissues.

There has been an emerging interest in the past few years for natural solvent-based essential oils, such as orange oil, peppermint oil, among others, as possible alternatives for the removal of gutta-percha. The reason for this is mainly attributed to the relatively good biocompatibility of these solvents along with their reduced cytotoxicity compared to the conventional solvents. As a result, they are deemed safe, especially concerning the reduced risk of periapical tissue damage (14). Natural solvents based on essential oils have been recommended as safer alternatives than the conventional gutta-percha solvents because of their relatively low toxicity levels. Nevertheless, their application is not entirely free from drawbacks. It has been revealed that these solvents tend to deposit thin layers on the dentin surfaces or cause changes in the chemical composition of the dentin. This may hinder the adhesion of sealers through the micromechanical interlocking process.

Consistent with the above, the current *in vitro* investigation was performed to evaluate the impact of different gutta-percha dissolvers, namely xylene, castor oil, peppermint oil, and wintergreen oil, on the bonding capacity of the AH Plus sealer to root dentin post-retreatment.

The results indicated that the choice of solvent influenced the push-out bond strength of AH Plus sealer to root dentin. Statistically significant differences were observed in several pairwise comparisons among the solvent groups, whereas no statistically significant difference was observed between the wintergreen oil and xylene groups.

In comparison with other solvents, castor oil provided the greatest bond strength followed by wintergreen oil, xylene, and peppermint oil. The superior bond strength observed with castor oil suggests that it may create a more favorable dentinal substrate for resin sealer adhesion compared to other solvents, though the exact surface interactions require further study.

These results present an interesting contrast to previous reports indicating that castor oil may leave more leftover gutta-percha residue in the canal compared to other solvents (15). While such residues typically interfere with sealer bonding, the superior performance of castor oil in this study may be attributed to its unique chemical composition. Castor oil is predominantly composed of ricinoleic acid, which can act as a powerful surfactant. We tentatively hypothesize that this property might act as a surfactant to enhance the wettability of the dentinal walls, potentially compensating for physical residue. However, this proposed mechanism is highly speculative, especially as it appears to contradict prior residue findings (15). Because this study lacks validation via scanning electron microscopy (SEM) or energy-dispersive x-ray spectroscopy (EDS), this explanation remains strictly theoretical.

However, peppermint oil exhibited the weakest bond strength compared to other oils evaluated in the current study. The lower bond strength observed with peppermint oil may potentially be related to differences in gutta-percha removal and/or alterations in the dentinal substrate. However, residual gutta-percha and dentinal surface characteristics were not directly evaluated in the present study; therefore, this proposed mechanism should be interpreted cautiously. Presence of gutta-percha particles would not allow the sealer to penetrate the canals, hence creating weak bonds with dentin (14, 16). Intermediate values were observed for wintergreen oil in terms of bond strength, and there was no statistically significant difference between it and xylene. Wintergreen oil is a methyl salicylate-based solvent that is known to dissolve gutta-percha within five minutes (17).

The gutta-percha solvent based on aromatic hydrocarbons xylene showed satisfactory bond strengths, although there were larger variations in its results. Despite the good solvency effect of xylene, and its lesser toxicity compared to chloroform, questions still exist about its influence on dentine. It was reported that prolonged exposure to xylene can lead to changes in the hardness and mineralization of dentine, which might negatively impact the bonding of resin sealers. In particular, xylene influences the organic component, resulting in the softening of dentine (10).

Findings from the current research are in conformity with earlier studies showing that the application of solvents can change the characteristics of dentine and thus influence the bonding characteristics of endodontic sealers (18–20). The findings on the efficacy of using natural oils like castor oil have provided further encouragement to consider natural substitutes for traditional gutta-percha dissolving solvents used in endodontic treatments.

While providing valuable insights, this *in vitro* study has several limitations. Methodologically, the total 4-week aging period (split across two separate 2-week storage phases) may not fully reflect the long-term clinical degradation of the sealer interface. Additionally, using a single root section per tooth and a standardized two-minute solvent application restricted the evaluation of continuous canal effects and slower dissolution kinetics. Because root-third and arch-origin variability were not treated as independent factors, pooling the coronal, middle, and apical sections introduced unmeasured within-group variance due to differing dentinal tubule densities. Re-obturating with the same sealer (AH Plus) yielded a combined bond strength outcome, preventing the isolation of exact failure mechanisms. Furthermore, complete gutta-percha removal was verified only subjectively (visual, tactile, and radiographic) rather than through advanced objective imaging like micro-CT, leaving the possibility of microscopic residue unaddressed. Finally, because the primary objective was assessing macroscopic mechanical retention, scanning electron microscopy (SEM) and failure mode classification were omitted, limiting comprehensive micro-structural evaluation. Future studies incorporating mixed-effects modeling, prolonged aging periods, varied kinetics, objective imaging, and detailed interfacial analyses are highly recommended.

## Conclusion

Considering the limitations of this *in vitro* study, the kind of solvent utilized in gutta-percha removal had an important bearing on the bond strength of the AH Plus sealer with the root dentin. The castor oil proved to yield the best bond strength, implying better retention and improved adhesion between the dentin and the sealer. On the other hand, the wintergreen oil was found to be equally effective as xylene, hence making it possible to use the former as an alternative to the traditional solvents. The peppermint oil had the least bond strength of the three oils evaluated.

Thus, the selection of the appropriate solvent is important since it plays an important role in the sealer-adhesion process.

## Data Availability

The original contributions presented in the study are included in the article/Supplementary Material, further inquiries can be directed to the corresponding author.
